# Incorrect Use of Face Masks during the Current COVID-19 Pandemic among the General Public in Japan

**DOI:** 10.3390/ijerph17186484

**Published:** 2020-09-06

**Authors:** Masaki Machida, Itaru Nakamura, Reiko Saito, Tomoki Nakaya, Tomoya Hanibuchi, Tomoko Takamiya, Yuko Odagiri, Noritoshi Fukushima, Hiroyuki Kikuchi, Shiho Amagasa, Takako Kojima, Hidehiro Watanabe, Shigeru Inoue

**Affiliations:** 1 Department of Preventive Medicine and Public Health, Tokyo Medical University, Tokyo 160-8402, Japan; machida@tokyo-med.ac.jp (M.M.); takamiya@tokyo-med.ac.jp (T.T.); odagiri@tokyo-med.ac.jp (Y.O.); fukufuku@tokyo-med.ac.jp (N.F.); kikuchih@tokyo-med.ac.jp (H.K.); amagasa@tokyo-med.ac.jp (S.A.); 2 Department of Infection Prevention and Control, Tokyo Medical University Hospital, Tokyo 160-0023, Japan; task300@tokyo-med.ac.jp (I.N.); hw-nabe4@tokyo-med.ac.jp (H.W.); 3 Division of International Health (Public Health), Graduate School of Medical and Dental Sciences, Niigata University, Niigata 951-8510, Japan; jasmine@med.niigata-u.ac.jp; 4 Graduate School of Environmental Studies, Tohoku University, Miyagi 980-0845, Japan; tomo.nakaya@gmail.com (T.N.); info@hanibuchi.com (T.H.); 5 Department of International Medical Communications, Tokyo Medical University, Tokyo 160-0023, Japan; tkojima@tokyo-med.ac.jp

**Keywords:** COVID-19, pandemic, face mask, protective measures, epidemiology, public health

## Abstract

Since the emergence of the COVID-19 pandemic, the use of face masks by healthy individuals for prevention has been attracting public attention. However, efficacy depends on proper usage. We set out to determine the prevalence of wearing masks to prevent COVID-19 and compliance with appropriate measures for the correct use of face masks among the general public in Japan where wearing medical masks is a “cultural” normality. This cross-sectional study was based on an internet-based survey completed by 2141 people (50.8% men, aged 20–79 years) who were selected among registrants of an Internet research company between 1 April and 6 April 2020. Participants were asked to indicate how often they wore masks for prevention and to what extent they practiced appropriate measures suggested by the World Health Organization. The prevalence of wearing masks was 80.9% and compliance rates with appropriate measures ranged from 38.3% to 83.5%. Only 23.1% complied with all recommendations. Compliance rates were overall low in men and persons with low household incomes. Our results, hence show that many citizens implement inaccurate measures when using face masks. Therefore, providing guidance on correct usage is essential when encouraging the use of face masks to prevent COVID-19.

## 1. Introduction

The coronavirus disease 2019 (COVID-19) has caused a global pandemic [1]. It is vital that the public implement personal protective measures in order to mitigate virus transmission, especially before a well-matched vaccine becomes available [2]. During the COVID-19 pandemic, the use of facemasks by healthy individuals, which is a personal protective measure, has been attracting a lot of public attention. A recent review reported that wearing masks could possibly prevent against the transmission of COVID-19 [3]. Studies have found that COVID-19 viral loads detected in asymptomatic patients were similar to those in symptomatic patients [4] and that COVID-19 can be transmitted from asymptomatic patients [5]. Based on these findings, certain researchers have pointed out that wearing face masks in public spaces, including by those who are infected but are asymptomatic and contagious, may be effective in preventing the transmission of the virus [6,7]. Japan is a country where people have a cultural habit of wearing medical masks, irrespective of the COVID-19 pandemic. A large number of Japanese people wear medical masks on a daily basis, especially during flu season [8]. Regarding the current COVID-19 pandemic, health authorities in Japan have indicated that wearing face masks may be effective in preventing viral infection in closed environments with poor ventilation; therefore, a large number of Japanese people have been wearing medical masks in public spaces from the beginning of the outbreak [9]. While Japan has long had a culture of wearing medical masks, the implementation status of mask-wearing behaviors and the extent to which masks are correctly used was unclear. Mask-wearing may be an effective way to prevent transmission among people, whether they are symptomatic or asymptomatic, however if they are worn incorrectly, this could, in fact, potentially contribute to the transmission of the virus [10]. In order to prevent viral transmission, it is of utmost importance that the general public manages how to wear, remove, and dispose face masks correctly. Therefore, the objective of this study was to clarify mask-wearing behaviors among the Japanese general public and to determine the extent to which face masks are correctly used during the current COVID-19 pandemic.

## 2. Materials and Methods

### 2.1. Study Sample and Data Collection

This was a cross-sectional study utilizing data obtained from a second wave survey of a longitudinal study, which was initially conducted to clarify the implementation of personal protective measures by the general Japan population during the COVID-19 pandemic. The study participants were recruited from the registrants of a Japanese Internet research service company called MyVoice Communication, Inc., which had approximately 1.12 million registered participants as of January 2020. In the first wave survey of the longitudinal study on 25 February 2020, we collected data from 2400 men and women aged 20 to 79 years (sampling by sex and 10-year age groups; 12 groups, *n* = 200 in each group) who were living across seven prefectures in the Tokyo metropolitan area (i.e., Tokyo, Kanagawa, Saitama, Chiba, Ibaraki, Tochigi, and Gunma), which is home to approximately 35% of the total Japanese population (total area: 32,433.4 km^2^; total population: 43,512,238 people as of January 2019). The Internet research service company randomly chose the potential respondents from these registered participants. The number of potential respondents in each stratified sample group was determined by dividing 200 by the response rate for the corresponding sociodemographic group. The response rate was then computed based on the results of past surveys conducted by the research company. The number of potential respondents was *n* = 8156; male: 20–29 years, *n* = 1302; 30–39 years, *n* = 691; 40–49 years, *n* = 447; 50–59 years, *n* = 415; 60–69 years, *n* = 420, 70–79 years, *n* = 443, female: 20–29 years, *n* = 1740; 30–39 years, *n* = 840; 40–49 years, *n* = 579; 50–59 years, *n* = 486; 60–69 years, *n* = 436; and 70–79 years, *n* = 357. Registrants were invited to participate in the survey by e-mail (*n* = 8156). The questionnaires were placed in a protected area of a website and potential respondents received a specific Uniform Resource Locator (URL) in their invitation email. When the number of participants who responded to the questionnaire voluntarily reached 200 in each group, no more responses were accepted from that group. Data were obtained from 2400 participants for the first wave survey. In the second wave survey, the company reached out to the same participants by email, requesting their participation in the second wave survey on 1 April (*n* = 2400). At the time of the survey, a surge in the number of laboratory-confirmed COVID-19 cases was confirmed in Tokyo. Directly following the completion of the survey, a state of emergency was declared in Japan [11]. The questionnaires were placed in a secured section of a website and potential respondents received a specific URL in their email. Participation was voluntary. Participants responded to the questionnaire by accessing the specified URL. The response cut-off date was 6 April. Reward points valued at 50 yen were provided as an incentive for participation (approximately 0.5 US dollars as of February 2020). This study was approved by the Ethics Committee of Tokyo Medical University, Tokyo, Japan (No: T2019–0234). Informed consent was obtained from all respondents.

### 2.2. Assessment of Mask-Wearing and Correct Use of Face Masks

Participants were asked about the frequency in which they wore face masks during the previous week to prevent COVID-19 and responded using a 4-point-Likert scale (1. “Always,” 2. “Sometimes,” 3. “Rarely,” or 4. “Never”). Regarding the correct use of face masks, participants gave a self-report on the implementation of the following recommendations based on the interim guidance issued by the World Health Organization (WHO) (Table 1: placing the mask carefully, ensuring it covers the mouth and nose, avoiding touching the mask, removing the mask using the appropriate technique, practicing hand hygiene after removal or touching the mask, replacing the mask when it becomes damp, not re-using the mask, and discarding the mask immediately after use) [10]. Participants responded to questions related to these recommendations on ways to correctly handle face masks by using a 4-point Likert scale (“Always careful,” “Sometimes careful,” “Rarely careful”, and “Never careful”).

### 2.3. Assessment of Sociodemographic Factors

Participants reported their sex, age, marital status (not married/married), working status (working/not working), living arrangement (with others/alone), smoking (smokers/non-smokers), past medical history (hypertension, diabetes, and respiratory disease), residential area (Tokyo/other), and seasonal influenza vaccination history (received/not received annually). Moreover, the research company provided categorized data on the following: educational attainment (university graduate or above/below) and household income level (<5 million yen/≥5 million yen).

### 2.4. Statistical Analysis

In this study, when a participant responded with 1 or 2 (“Always”/“Sometimes”) on the 4-point-Likert scale, it was determined that wearing masks to prevent COVID-19 was implemented. Regarding the correct use of face masks, when a participant responded with 1 or 2 (“Always careful” or “Sometimes careful”) on the 4-point Likert scale, it was determined that the respondent was using face masks correctly. We clarified the prevalence of wearing masks to prevent COVID-19, the rate of compliance with each appropriate measure for correctly using face masks, and the proportion of those who practice all recommended measures.

To clarify the association between each sociodemographic factor and correct use of face masks, a multivariate logistic regression analysis was performed. The dependent variables consisted of each of the seven recommended measures for correct use of face masks and implementing all seven practices. Independent variables included sex, age (20–29/30–39/40–49/50–59/60–69/70–79 years), marital status (not married/married), working status (working/not working), living arrangement (with others/alone), smoking status (smokers/non-smokers), residential area (Tokyo/other), educational attainment (university graduate or above/below), and household income level (<5 million yen/≥5 million yen). In the sensitive analysis, an ordinal logistic regression analysis using a 4-point Likert scale with each appropriate measure for correctly using face masks was performed.

Statistical analyses were performed using IBM SPSS Statistics for Windows, version 26 (IBM Japan, Tokyo, Japan). Two-sided *p* values less than 0.05 were considered to be statistically significant.

## 3. Results

### 3.1. Participants Characteristics and Prevalence of Wearing Masks

Among the 2400 potential respondents, 2141 were valid responses. Table 2 shows the participant characteristics and the prevalence of wearing masks to prevent COVID-19, which was 80.9%.

### 3.2. Compliance Rates with Each Appropriate Measure for Correctly Using Face Masks

**Figure 1:**
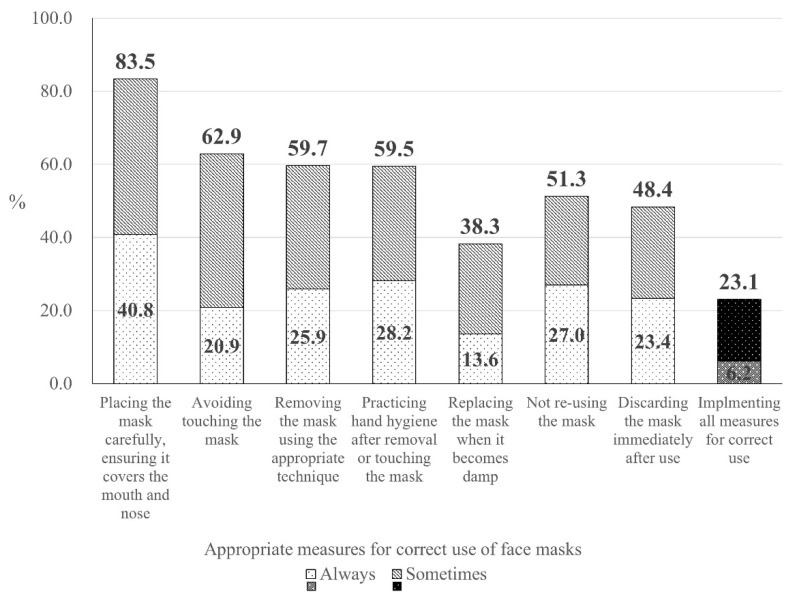
The prevalence of correct use of face masks. When a participant responded with 1 or 2 (“Always careful” or “Sometimes careful”) on the 4-point-Likert scale, it was determined that the respondent was using face masks correctly.

Figure 1 shows the compliance rate for each appropriate measure for correctly using face masks. We found that 83.5% of respondents were wearing their masks carefully, ensuring it covers the mouth and nose. However, compliance rates for other items ranged from 38.3% to 62.9%. Results indicated that 23.1% of respondents were in compliance with all appropriate measures for correctly using face masks.

### 3.3. Association Between Each Sociodemographic Factor and the Correct Use of Face Masks

Table 3 demonstrates the association between each sociodemographic factor and the correct use of face masks. According to multivariate logistic regression analysis, women and individuals with high household incomes generally had a significantly higher odds ratio than men and individuals with lower household incomes. In the sensitivity analysis using ordinal logistic regression analysis, the results were similar to those of the multiple logistic regression analysis, in which women and individuals with high household incomes generally had a significant positive association with correct use of face masks (Table S1). In both multivariate and ordinal logistic regression analyses, participants in their 50s and 60s revealed a negative association with “not re-using masks and discarding masks immediately after use”.

When a participant responded with 1 or 2 (“Always careful” or “Sometimes careful”) on the 4-point-Likert scale, it was determined that the respondent was using face masks correctly.

Multivariate logistic regression analysis was performed. Dependent variables included each measure for correctly using face masks and implementing all seven. Independent variables included sex, age (20–29/30–39/40–49/50–59/60–69/70–79 years), marital status (not married/married), working status (working/not working), living arrangement (with others/alone), smoking status (smokers/non-smokers), residential area (Tokyo/other), educational attainment (university graduate or above/below), and household income level (<5 million yen or ≥5 million yen).

## 4. Discussion

We set out to determine the prevalence of wearing face masks among the Japanese general public and compliance rates with appropriate measures for correctly using face masks. This study shed light on the following aspects of mask-wearing behaviors during the current COVID-19 pandemic: the percentage of Japanese people using face masks to prevent COVID-19 and the extent to which face masks are being correctly used. The prevalence of using face masks to prevent COVID-19 was approximately 80%. Meanwhile, the percentage of those who were in compliance with appropriate measures recommended by the WHO ranged from 38.3% to 83.5%. Only 23.1% of participants were practicing all measures. Furthermore, correct use of face masks was found to be lower among men and individuals with low household income levels than among women and individuals with high household income levels. This study found that the general public in Japan, despite having a cultural habit of wearing medical masks in daily life, especially during flu season, were using them incorrectly.

A recent study by Elachola et al. on the prevalence of wearing masks during the COVID-19 pandemic in international airports in four countries reported that the mask-wearing rate in France and the United States was 2–4% and 27% in Peru, while in Thailand, it was 46% [12]. Another study by Lee et al. reported that the prevalence of wearing masks in South Korea was 63.2% in February 2020 [13]. In many Asian countries, face masks are routinely used by the general public. However, the results from our study indicate that the use of masks among Japanese people may be particularly high, even among Asian countries where the mask-wearing rate is high. We recently reported that the prevalence of personal protective measures, such as hand hygiene, respiratory etiquette, and social distancing in Japan, was approximately 60% to 85% during the current COVID-19 outbreak [14]. Findings from this present study suggest that many Japanese are also using face masks to prevent COVID-19 in addition to simultaneously practicing personal protective measures mentioned above. It is possible that Japanese people voluntarily choose to wear face masks for preventive purposes during a respiratory virus pandemic, such as COVID-19, as they have become accustomed to do so [8].

This study evaluated the percentage of those who complied with each appropriate measure for correct use of face masks recommended by the WHO and found that compliance rates ranged from 38.3% to 83.5%. Only 23.1% of respondents undertook all WHO-recommended practices. In particular, compared to other appropriate measures for correctly using face masks, only a small percentage were replacing masks with new ones when they became damp. One reason for this may be due to global mask shortages [15]. Due to the current COVID-19 pandemic, the demand for face masks has increased up to 100-fold and prices have increased 20 times [16]. Japan has also been experiencing serious face mask shortages in communities and health care settings. In order to encourage people to practice the correct use of face masks, maintaining a stable supply is essential. Meanwhile, the following measures can be implemented regardless of mask shortages: placing the mask carefully, ensuring it covers the mouth and nose; avoiding touching the mask; removing the mask using the appropriate technique; and practicing hand hygiene after removing or touching the mask. While many Japanese commonly wear face masks, irrespective of the current pandemic, the prevalence of practicing these appropriate measures was insufficient. While the Japanese Ministry of Health, Labour, and Welfare has been conducting educational campaigns on the Internet about the correct use of masks [17], public awareness may be low. Although it is difficult to determine whether this prevalence among the Japanese general public is high or low compared to other countries, it is clear that further education is needed. Since compliance rates were overall low in men and individuals with low household incomes and people in their 50s and 60s were found to be re-using masks and not discarding masks immediately after use, it may be effective to provide education to target populations with such sociodemographic characteristics during a pandemic when time and resources may be limited. As of July 2020, the effectiveness of wearing face masks to prevent COVID-19 remains controversial. However, if health authorities are to recommend this practice, it is important to educate the general public on the correct use of face masks.

There are some limitations that should be considered in our study. The foremost point is the fact that participants were recruited among people registered in a single Internet research company and very little is known about online communities and the characteristics of people involved [18]. According to the latest White Paper 2019 issued by the Japanese government [19], the percentage of those who use the Internet regularly were approximately 95% for people in their 20s to 50s and 76.6% and 51.0% for those in their 60s and 70s, respectively. In addition, it has been reported that individuals who use the Internet regularly tend to have higher income, compared to non-users [19]. Therefore, it can be said that participants in this study may be more educated or have a higher income than the average of the Japanese population. Additionally, when comparing the age and sex composition of participants in this study to the latest population estimates reported by the Japanese government [20], there were more people in their 20s and fewer in their 40s in this study. Furthermore, there were 259 non-responders in the second wave survey. Many of the non-responders were women and non-smokers (data not shown). Considering these facts, the results of this study, such as percentages for mask use prevalence and compliance, should be interpreted with caution as they may have been affected by selection bias and may be overestimated. Second, we did not ask the participants whether they used medical masks or non-medical masks. The use of masks is recommended, however the advice that is given by the WHO on proper usage differs slightly between medical and non-medical masks [10]. Since Japanese people have been accustomed to using medical masks [8], it is very likely that a large number of participants in this study may have used a medical mask. Third, this study was a self-reported evaluation and these results may be affected by recall bias [21]. There may be a discrepancy between people’s perception of the correct use of face masks and actual behavior. Additionally, it is possible that social desirability bias may have led to an overestimation of the implementation status [22]. Finally, the results may not be applicable to other population groups. In populations with different cultural, ethnic, and geographical backgrounds, the frequency and number of hand hygiene events may vary greatly when compared with those reported in the present survey. Despite these limitations, to the best of our knowledge, this study is the first of its kind that has clarified mask-wearing behaviors and the compliance status of the correct use of face masks in Japan during the current COVID-19 pandemic.

## 5. Conclusions

The prevalence of wearing face masks to prevent COVID-19 among the Japanese general public was approximately 80%. Our results indicate that rates of compliance with appropriate measures for correct face mask usage recommended by the WHO ranged from 38.3% to 83.5% and that only 23.1% of participants were in compliance with all recommended measures. When encouraging the general public to use face masks, it is vitally important to ensure a stable supply and to provide accurate guidance on ways to correctly use face masks.

## Figures and Tables

**Table 1:** Recommendations on the correct use of face masks according to the interim guidance issued by the World Health Organization ^1^.

	Recommendations	How to Refer in this Study
1	Place the mask carefully, ensuring it covers the mouth and nose, and tie it securely to minimize any gaps between the face and the mask.	Placing the mask carefully, ensuring it covers the mouth and nose.
2	Avoid touching the mask while wearing it.	Avoiding touching the mask.
3	Remove the mask using the appropriate technique: do not touch the front of the mask but untie it from behind.	Removing the mask using the appropriate technique.
4	After removal or whenever a used mask is inadvertently touched, clean hands using an alcohol-based hand rub or soap and water if hands are visibly dirty.	Practicing hand hygiene after removing or touching the mask.
5	Replace masks as soon as they become damp with a new clean, dry mask.	Replacing the mask when it becomes damp.
6	Do not re-use single-use masks.	Not re-using the mask.
7	Discard single-use masks after each use and dispose of them immediately upon removal.	Discarding the mask immediately after use.

^1^ We referred to the interim guidance from the World Health Organization [10].

**Table 2:** Participant characteristics and the prevalence of wearing face masks to prevent COVID-19.

	*n* = 2141	
	*n* (%)/ Mean (SD ^1^)	
Sex (men)	1087	(50.8%)
Age (years)	49.7	(16.2)
Marital status (married)	1216	(56.8%)
Working status (working)	1352	(63.1%)
Living arrangement (with others)	1695	(79.2%)
Smoking (smokers)	318	(14.9%)
Past medical history (yes)		
Hypertension	398	(18.6%)
Diabetes	125	(5.8%)
Respiratory disease	91	(4.3%)
Residential area (Tokyo)	824	(38.5%)
Educational attainment (University graduate or above);	1122	(52.4%)
Household income level (≥5 million yen)	1101	(51.4%)
History of seasonal influenza vaccination (received annually)	585	(27.3%)
The prevalence of wearing face masks to prevent COVID-19 ^2^	1731	(80.9%)

^1^ Standard deviation. ^2^ When a participant responded with 1 or 2 (“Always”/ “Sometimes”) on the 4-point-Likert scale, it was determined that wearing face masks to prevent COVID-19 was implemented.

**Table 3:** Association between each sociodemographic factor and correct use of face masks.

	Placing the Mask Carefully, Ensuring it Covers the Mouth and Nose	Avoiding Touching the Mask	Removing the Mask Using the Appropriate Technique	Practicing Hand Hygiene after Removal or Touching the Mask	Replacing the Mask when it Becomes Damp	Not Re-Using the Mask	Discarding the Mask Immediately after Use	Implementing all Measures for Correct use of Face Masks
*n* = (2141)	Odds Ratio (95% Confidence Interval)							
Sex:								
Men	Ref.^1^	Ref.	Ref.	Ref.	Ref.	Ref.	Ref.	Ref.
Women	2.02 ** (1.56–2.63)	1.67 ** (1.37–2.04)	1.38 * (1.13–1.67)	1.63 ** (1.34–1.98)	1.35 * (1.11–1.65)	1.38 * (1.14–1.68)	1.61 ** (1.33–1.96)	1.30 * (1.04–1.64)
Age:								
20–29	Ref.	Ref.	Ref.	Ref.	Ref.	Ref.	Ref.	Ref.
30–39	0.92 (0.60–1.40)	0.87 (0.62–1.22)	0.94 (0.68–1.31)	1.01 (0.72–1.41)	0.97 (0.70–1.36)	0.97 (0.70–1.35)	0.73 (0.53–1.02)	0.95 (0.66–1.37)
40–49	1.27 (0.81–1.99)	0.80 (0.57–1.13)	0.76 (0.54–1.06)	1.12 (0.79–1.57)	0.77 (0.55–1.09)	0.70 * (0.50–0.98)	0.66 * (0.47–0.93)	0.63 * (0.43–0.93)
50–59	1.09 (0.69–1.71)	0.97 (0.68–1.39)	0.98 (0.70–1.39)	0.88 (0.62–1.24)	0.77 (0.54–1.09)	0.58 * (0.41–0.81)	0.50 ** (0.36–0.71)	0.65 * (0.44–0.97)
60–69	1.27 (0.79–2.02)	0.77 (0.54–1.11)	0.97 (0.68–1.38)	0.83 (0.58–1.18)	0.92 (0.65–1.32)	0.64 * (0.45–0.90)	0.50 ** (0.35–0.70)	0.69 (0.46–1.03)
70–79	1.31 (0.79–2.17)	1.07 (0.73–1.58)	1.06 (0.73–1.55)	1.00 (0.68–1.45)	1.41 (0.97–2.05)	0.83 (0.58–1.21)	0.60 * (0.42–0.87)	0.84 (0.55–1.28)
Marital Status:								
Not Married	Ref.	Ref.	Ref.	Ref.	Ref.	Ref.	Ref.	Ref.
Married	0.89 (0.65–1.24)	1.03 (0.81–1.32)	0.83 (0.65–1.06)	0.85 (0.66–1.08)	0.98 (0.77–1.26)	0.84 (0.66–1.06)	0.90 (0.71–1.14)	0.90 (0.68–1.19)
Working Status:								
Not Working	Ref.	Ref.	Ref.	Ref.	Ref.	Ref.	Ref.	Ref.
Working	0.88 (0.65–1.19)	0.85 (0.68–1.07)	0.89 (0.71–1.11)	0.89 (0.71–1.11)	1.01 (0.81–1.26)	1.20 (0.96–1.49)	1.29 * (1.04–1.61)	1.04 (0.80–1.34)
Living Arrangement:								
Alone	Ref.	Ref.	Ref.	Ref.	Ref.	Ref.	Ref.	Ref.
with Others	1.10 (0.77–1.56)	1.18 (0.89–1.55)	1.29 (0.98–1.70)	1.32 (1.00–1.74)	1.01 (0.76–1.34)	1.19 (0.90–1.56)	1.22 (0.92–1.60)	1.21 (0.87–1.68)
Smoking:								
Non-Smoker	Ref.	Ref.	Ref.	Ref.	Ref.	Ref.	Ref.	Ref.
Smoker	0.90 (0.65–1.24)	0.90 (0.70–1.16)	1.04 (0.80–1.34)	1.09 (0.84–1.40)	1.32 * (1.02–1.71)	1.38 * (1.07–1.77)	1.27 (0.98–1.63)	1.10 (0.82–1.49)
Residential Area:								
Tokyo	0.82 (0.64–1.04)	0.97 (0.80–1.17)	0.99 (0.82–1.19)	0.98 (0.81–1.18)	0.82 * (0.68–1.00)	1.07 (0.89–1.29)	0.98 (0.82–1.18)	0.85 (0.68–1.06)
Others	Ref	Ref.	Ref.	Ref.	Ref	Ref.	Ref.	Ref.
Educational Attainment:								
University Graduate or Above	0.94 (0.73–1.22)	0.96 (0.79–1.17)	0.92 (0.76–1.11)	1.09 (0.90–1.32)	0.91 (0.75–1.11)	1.03 (0.85–1.24)	1.10 (0.91–1.32)	1.08 (0.86–1.35)
Below University Level	Ref.	Ref.	Ref.	Ref.	Ref.	Ref.	Ref.	Ref.
Household Income:								
<5 Million Yen	Ref.	Ref.	Ref.	Ref.	Ref.	Ref.	Ref.	Ref.
≥5 Million Yen	1.70 ** (1.30–2.22)	1.21 (0.99–1.49)	1.30 * (1.07–1.59)	1.27 * (1.04–1.55)	1.29 * (1.05–1.58)	1.39 * (1.14–1.69)	1.22 (1.00–1.48)	1.17 (0.92–1.47)

^1^ Ref.: Reference; *p* value; * : <0.05, ** : <0.001.

## Supplementary Materials

The following are available online at https://www.mdpi.com/1660-4601/17/18/6484/s1, Table S1: association between each sociodemographic factor and correct use of face masks for ordinal logistic regression.
