# Supplementary figures and images for: Incorrect Use of Face Masks during the Current COVID-19 Pandemic among the General Public in Japan
Source: Int J Environ Res Public Health. 2020 Sep 6;17(18):6484. doi: 10.3390/ijerph17186484 (PMC7557398; doi:10.3390/ijerph17186484)

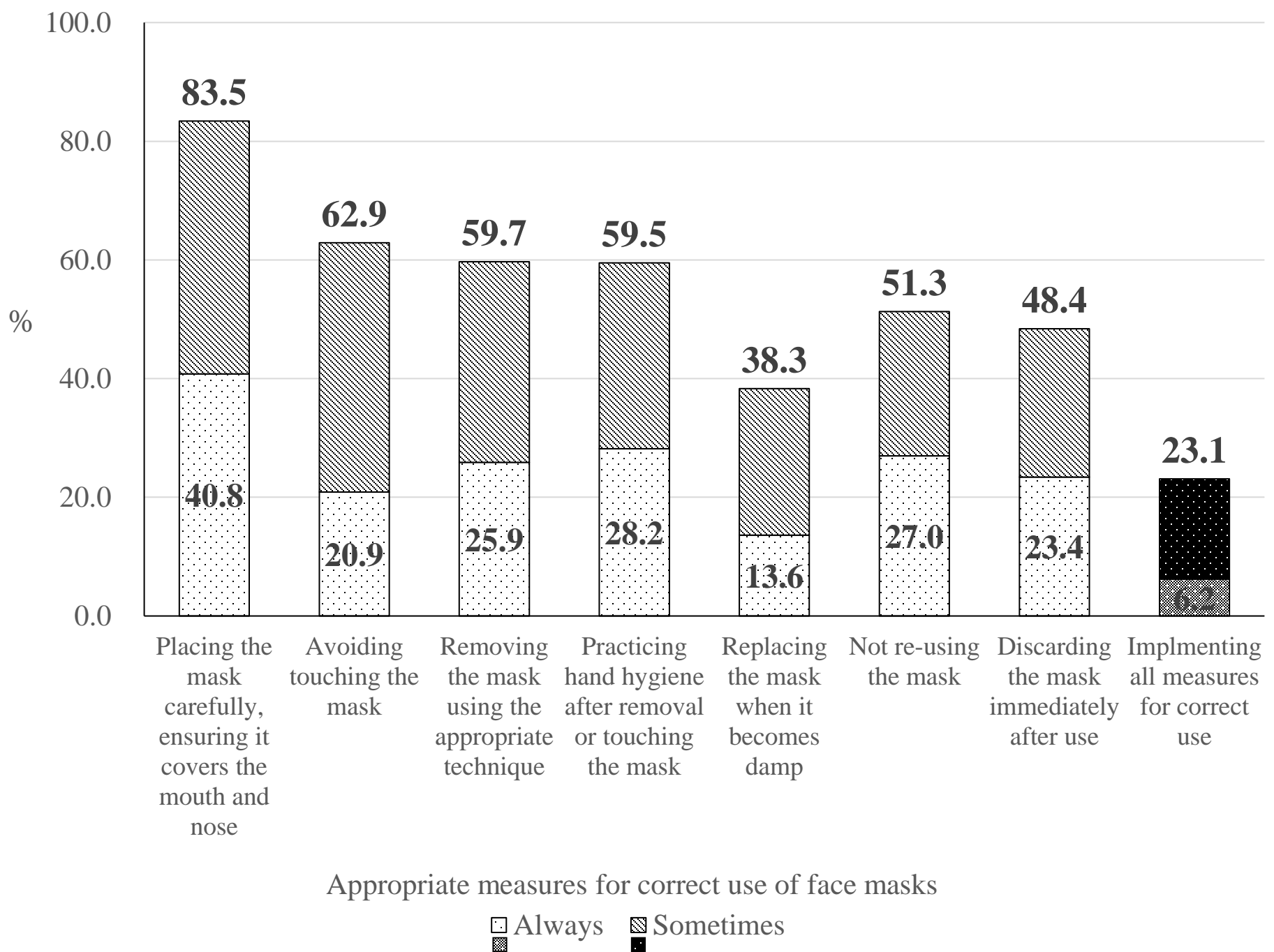

Supplement: Supplementary file 1 [file ijerph-17-06484-s001.zip › Figure 1_revised.pdf]
